# The Food and Nutritional Diseases of an Army

**Published:** 1917-11-24

**Authors:** 


### THE FOOD AND NUTRITIONAL DISEASES OF AN ARMY.

### How the British Soldier is Cared For.

This was the subject of the concluding Chadwick Lecture delivered on November 14 by Dr. Woods Hutchinson on the part played by hygiene in the European war. Dr. Hutchinson said this terrible war had placed the doctor really in control of the springs which made for the health and wholesomeness of that large part of the community which had been drafted into military service. A tribute to the fine sanitary work of the British Army was thus expressed by an American officer: "If we can get anything like as good as that inside five years, we shall consider ourselves very lucky." Every man who fell wounded on the field of battle could now ensure getting as good treatment, and quickly, as if he were a millionaire in a country seat. As a rule, within eight hours of receiving his wound he was in a comfortable bed and being nursed in a most efficient manner.

### Food More Essential than Drugs.

The subject of his lecture that day was a consideration of the food problem, and the diseases attributable to peculiarities, defects, or deficiencies of food. The more the question was investigated, the more was the attention given to food and the less to drugs. With the exception of infections and their treatment by specific vaccines, disease was only permanently cured by food: good feeding and plenty of it was the great mainstay. The foods supplied to the soldiers on the Western front were magnificent: they needed to be seen to be appreciated. They had been planned by physiological experts so that they might meet the demands of the soldier. In the doctor's work among fighting men, drugs played a very small part. A great variety of foods were required to supply the needs of the body, each having its special function, but if he were asked what food stood out pre-eminently in its building and sustaining qualities for armies, he would say an abundant supply of meat. The majority of the soldiers on the Western front received meat two or three times a day, and there was also a generous supply of fresh vegetables. He would like to express his high appreciation of the work of the Army cooks and rationing parties, for the success with which they brought up supplies to men in advanced positions often amounted to real heroism. Sometimes a good proportion of the wounded men who came trickling back belonged to these rationing parties. He was able to praise the soldiers' food because he had lived on it himself, and its meat content was richer than many of the men had been accustomed to at home. To hear vegetarians talk, one would have expected large numbers of these men, eating so much meat and living in wet and bad-weather conditions, would have been badly crippled with rheumatism, gout, arterio-sclerosis, and conditions attributed by large-type advertisements to uric acid. But the fact was that these metabolic diseases were significantly absent.

### The Importance of Good Bread.

The next important element in the British soldier's food was the abundant supply of good bread. The generosity of the whole dietary could be judged from the fact that the basis of provision was five tons of food for every thousand men. The bread which had the highest proportion of nutriment was the so-called "white bread." Faddists had pronounced it to be anæmic and mineralless, but faddists were never wholly right, and it was always safe to make a 50-per-cent. discount when listening to their assertions. One result of the wholemeal propaganda had been to increase immensely the ratio of profits gained by "Health-food" vendors. Certainly one of the effects of wholemeal bread was to cause increasing intestinal irritation, sometimes gastritis. There was no need for a scare about shortage of bread, as there were two years' supplies in Australia, only waiting to be fetched, and he had no sympathy with the stipulation that bread must have been kept twenty-four hours before it was allowed to be used; it was very short-sighted economy. The people of the nation, as a whole, did not eat too much; that they did so was the idea of arm-chair clubmen. The Health Commission which had to inquire into the welfare of munition workers sampled the foods taken by the workers for their meals by meeting a certain number and purchasing their dinner-baskets and their contents, afterwards subjecting them to analysis; and on the average the food taken in came within 10 per cent. of the calories needed to do hard bodily work.

### The Soldier Needs Sugar.

A third important element in the food supply of an army was an abundant supply of sugar, jam, or other sweet substances. Sugar was not only one of the most digestive foods we have, but it was one of the quickest to be converted into actual energy. American estimates in peace-time were based upon 1 lb. per person per day. The free use of sugar was very important, and the soldier was keen upon chocolate not only because it was sweet, but because he needed it. It must never be forgotten that the vigorous out-door life of the soldier generally ensured a complete utilisation of all the value in the foods he took; hence the ailments ascribable to the clogging by waste products were very rare among soldiers. Among the men in the trenches the percentage of cases of rheumatism, influenza, bronchitis, and even sore throat was less than among the general population of London or of Paris. So also in the case of intestinal trouble: diarrhœa, dysenteries, gastritis; all were quite rare at the Front. That was probably largely due to the care with which the foods were handled, thus lessening the chance of infection from without; it had been a great pleasure to him to see how the food was handled at every stage of its course. In every camp there was the danger arising from the setting up of a vicious circle of inoculation, starting from the "carrier," the person who, having had a disease, and recovered from the symptoms, still carried the germs of it about with him, and was thus liable to infect food. He spoke of the improvement in the kind of latrine used, with the tremendously lessened risk of fly contamination. It was very difficult to convince the French and Belgian peasant that the manure-heap at the entrance to a homestead was a source of disease, but a strong effort had been made to rid the vicinity of camps of garbage heaps and all such wholesale disease-breeders. Much of the water, too, in the Western part of Europe not only smelt but tasted of sewage, and in this regard also much difficulty had had to be overcome. Protection against fly contamination the lecturer found more thoroughly carried out on the Italian than on any of the other Western fronts, and this had been much helped by the dry climate, for refuse was soon dried sufficiently to be burned.

### THE FOOD AND NUTRITIONAL DISEASES OF AN ARMY—(*continued*).

Varying Incidence of Consumption.

The greatest scourge of civil life was consumption, which depended on insufficient food and over-crowded living, sleeping, and working rooms. The only methods in which the profession had confidence, in addition to tuberculin in certain classes of case, were an abundance of good nourishing food and life in the open air. The dug-outs and places into which the soldier was driven for shelter did not conduce to the diminution of tuberculosis infection; still, those underground shelters were no worse than the conditions under which many working people had perforce to sleep in peace-times; certainly they were not worse than the conditions in which the French and Italians lived in the ordinary way, for they sealed up their doors and windows, and seemed to have become adept at "consuming their own smoke." At first there was a diminution of consumption on our Front, but now there was a slight increase. The explanation given was that a fair proportion of men in the Army had at some time had tuberculosis. Their open-air life resulted in an improved physique and resistance against tubercle bacilli. But with the strain of war there came a time when that resistance gave out, and they were again sufferers from their old complaint. This second current was now becoming obvious, and the Army authorities were concerning themselves with the provision of suitable hospitals for these men.

To be at the Front induced the belief that at last there had appeared upon the earth a great industrial democracy, a great community of effort, a Utopia in which the interests and efforts of each were for the whole, and he believed that when the men came back from their marvellous experience of the effectiveness of human comradeship, the world would be found to have been lifted to a higher plane of kindness and justice.

